# Different dissecting orders of the pulmonary bronchus and vessels during right upper lobectomy are associated with surgical feasibility and postoperative recovery for lung cancer patients

**DOI:** 10.1186/s40880-017-0220-9

**Published:** 2017-06-27

**Authors:** Hao-Ran Zhai, Xue-Ning Yang, Qiang Nie, Ri-Qiang Liao, Song Dong, Wei Li, Ben-Yuan Jiang, Jin-Ji Yang, Qing Zhou, Hai-Yan Tu, Xu-Chao Zhang, Yi-Long Wu, Wen-Zhao Zhong

**Affiliations:** 1 Graduate School, Southern Medical University, Guangzhou, Guangdong 510515 P. R. China; 2 Guangdong Lung Cancer Institute, Guangdong General Hospital & Guangdong Academy of Medical Sciences, Guangzhou, Guangdong 510080 P. R. China

**Keywords:** Lung cancer, Dissecting order, Video-assisted thoracic surgery, Pulmonary veins, Bronchus

## Abstract

**Background:**

Right upper lobectomy (RUL) for lung cancer with different dissecting orders involves the most variable anatomical structures, but no studies have analyzed its effects on postoperative recovery. This study compared the conventional surgical approach, VAB (dissecting pulmonary vessels first, followed by the bronchus), and the alternative surgical approach, aBVA (dissecting the posterior ascending arterial branch first, followed by the bronchus and vessels) on improving surgical feasibility and postoperative recovery for lung cancer patients.

**Methods:**

According to the surgical approach, consecutive lung cancer patients undergoing RUL were grouped into aBVA and VAB cohorts. Their clinical, pathologic, and perioperative characteristics were collected to compare perioperative outcomes.

**Results:**

Three hundred one patients were selected (109 in the aBVA cohort and 192 in the VAB cohort). The mean operation time was shorter in the aBVA cohort than in the VAB cohort (164 vs. 221 min, *P* < 0.001), and less blood loss occurred in the aBVA cohort (92 vs. 141 mL, *P* < 0.001). The rate of conversion to thoracotomy was lower in the aBVA cohort than in the VAB cohort (0% vs. 11.5%, *P* < 0.001). The mean duration of postoperative chest drainage was shorter in the aBVA cohort than in the VAB cohort (3.6 vs. 4.5 days, *P* = 0.001). The rates of postoperative complications were comparable (*P* = 0.629). The median overall survival was not arrived in both cohorts (*P* > 0.05). The median disease-free survival was comparable for all patients in the two cohorts (not arrived vs. 41.97 months) and for patients with disease recurrences (13.25 vs. 9.44 months) (both *P* > 0.05). The recurrence models in two cohorts were also comparable for patients with local recurrences (6.4% vs. 7.8%), distant metastases (10.1% vs. 8.3%), and both (1.8% vs. 1.6%) (all *P* > 0.05).

**Conclusions:**

Dissecting the right upper bronchus before turning over the lobe repeatedly and dissecting veins via the aBVA approach during RUL would promote surgical feasibility and achieve comparable postoperative recovery for lung cancer patients.

**Electronic supplementary material:**

The online version of this article (doi:10.1186/s40880-017-0220-9) contains supplementary material, which is available to authorized users.

## Background

Lung cancer is the leading cause of cancer-related deaths worldwide [1], especially in China [2]. Several studies have indicated promising perioperative outcomes in patients with early-stage non-small cell lung cancer (NSCLC) requiring standardized surgery [3–6] or complicated surgery [7–9]. Video-assisted thoracoscopic surgery (VATS) has also been well established [10–12] and validated by a propensity score analysis [13]. Because of the rapid development of VATS techniques, the surgical approaches used during open thoracotomy can be applied to VATS; but some surgical procedures need further improvements to facilitate VATS owing to its different views of the thorax, the broadened surgical field, and other reasons. Using VATS, certain alternative dissecting orders would create differences in surgical feasibility compared with conventional surgical procedures. Coupled with specific anatomical structures of each lobe, the dissecting order of the pulmonary bronchus, veins, and arteries during VATS may play a vital role in promoting postoperative recovery and prolonging survival. However, no studies have analyzed how the different orders of dissecting the pulmonary vessels and the bronchus affect surgical feasibility and disease recurrences. As reported in previous works, most primary lung cancers (29.2%–37.2%) occur in the right upper lobe, and right upper lobectomy (RUL) involves the most complicated hilar anatomical structures and requires various surgical procedures [10, 14–16]. Analyzing the effects of different dissecting orders among homogeneous patients requiring RUL would provide more insights about what factors should be paid more emphasis to improve postoperative recovery and prolong survival.

Various principles have been proposed to facilitate surgical manipulations. Liu et al. [8] reported the single-direction lobectomy approach, which was described as dissection starting at the root of the hilus and proceeding gradually from the most superficial to deeper structures in a single direction, that is, from the right upper veins/arteries to the right upper bronchus (the VAB approach). Fused fissures were transected before removing the resected lobe from the chest, thereby shortening the duration of air leakage and reducing the risk of vascular damage; this “fissureless technique” has potentially expanded indications for VATS [7, 9, 17].

Subsequently, Yan [18] summarized a reliable and reproducible technique called the posterior approach. This procedure started with the dissection of the posterior ascending artery, facilitated access to the posterior hilus, and provided a clear vision of the hilar and interlobar lymph nodes, which would be especially appropriate for video-assisted thoracoscopic segmentectomy. However, traditionally, it was highly suggested that the pulmonary veins should be interrupted first to avoid dissemination of tumor cells [19–21]. Circulatory tumor cells are detectable in the pulmonary veins and would lead to more distant metastases and treatment failure [20]. However, Refaely et al. [22] concluded that dissecting either the veins or the arteries first would not interfere with disease recurrence or survival. Therefore, the dissecting order of pulmonary structures remains controversial.

These studies were all based on conventional thoracotomy instead of VATS and involved lesions distributed in different lobes. Bias in the heterogeneity of patients and lobectomies would interfere with appropriate interpretation of existing data. Because VATS is widely acknowledged and used for lobectomy, it is quite necessary to identify the advantages and disadvantages of conventional (VAB) and alternative approaches, as well as how they affect surgical feasibility and postoperative recovery. Taking the anatomical structures of the right upper lobe into consideration, we speculated that different dissecting orders of the pulmonary bronchus, veins, and arteries via VATS would affect technical safety and disease recurrence. In this study, we selected lung cancer patients who underwent RUL to compare their surgical feasibility, postoperative recovery, survival, and recurrence models so as to evaluate the surgical feasibility and long-term benefits of different orders of dissecting pulmonary structures during RUL.

## Patients and materials

### Study cohorts

Clinical data of all patients diagnosed with lung cancers who were referred to Guangdong General Hospital & Guangdong Lung Cancer Institute (Guangzhou, China) between January 2013 and April 2015 were screened through the electronic medical record system. This study was approved by the Ethics and Scientific Committees of Guangdong General Hospital, and written informed consent forms were signed by all patients. Inclusion criteria were as follows: (1) primary lung cancers treated with RUL and (2) RUL intended to be performed via VATS, including those procedures that were converted to open thoracotomy during surgery. Exclusion criteria were as follows: (1) lung cancers on lobes other than the right upper lobe or (2) lung cancers on the right upper lobe treated with wedge resections, bilobectomy, pneumonectomy, or any other partial/expanded surgery.

Histological subtypes were determined according to the 2015 World Health Organization (WHO) Classification of Lung Tumors [23], and pathologic or clinical staging was based on the seventh edition of the International Association for the Study of Lung Cancer (IASLC) Tumor-Node-Metastasis (TNM) Staging Project [24]. Some advanced cases with intrathoracic dissemination or malignant pleural nodules (M1a) were accidently identified during surgery. As analyzed in previous studies [25] and according to the data of our institute (not published), these M1a patients may benefit from radical resection of the primary tumor lesion and subsequent systemic regimens. These patients with advanced disease receiving surgeries in this study were diagnosed accidentally during surgery. Both patients with early-stage lung cancer and those with accidently diagnosed advanced disease were selected for the analysis.

### Perioperative variables

We analyzed some variables related to surgical difficulties, including the maximum diameter and stage of the lesion, the number of dissected lymph nodes (LN) and LN stations, and the presence of fused fissures and intrathoracic adhesion.

Intraoperative variables, including the rate of intraoperative conversion to thoracotomy and corresponding reasons, were analyzed to evaluate surgical feasibility. Reasons for the conversion were classified into two groups: (1) VATS-related reasons, such as vessel injuries, which could be potentially caused by the limited surgical field or VATS procedures and be overcome by thoracotomy; and (2) non-VATS-related reasons, which were associated with intrinsic difficulties mostly due to disease-related anatomical malformations, such as intrathoracic adhesion and dense hilar and interlobar lymphadenectomy. Sometimes the conversion was caused by several reasons; therefore, we evaluated each surgical record to identify the direct reason that made surgeons decide to do the conversion and analyzed the data to explore the potential surgical feasibility and safety.

Postoperative variables, including duration of postoperative chest drainage, postoperative hospitalization, and rates of postoperative complications, were analyzed to compare short-term benefits. Postoperative complications were carefully reviewed and categorized as follows: (1) procedure-specific surgical complications caused by VATS manipulations and (2) non-procedure-specific medical complications resulting from preoperative respiratory, cardiac, or other system diseases.

### Surgical approaches

Eligible patients were grouped according to the dissecting order: dissecting the posterior ascending branch first, followed by the right upper bronchus and pulmonary vessels (aBVA); or taking the conventional VAB approach. Specifically, for patients with completely fused oblique fissures, VAB would be chosen because it was more feasible to proceed from the superficial veins toward the deeper arteries and bronchus. However, aBVA was also optimal if the right upper bronchus could be exposed by opening the posterior mediastinal pleura. With other conditions, the surgical approaches were decided by surgeons.

**Fig. 1:**
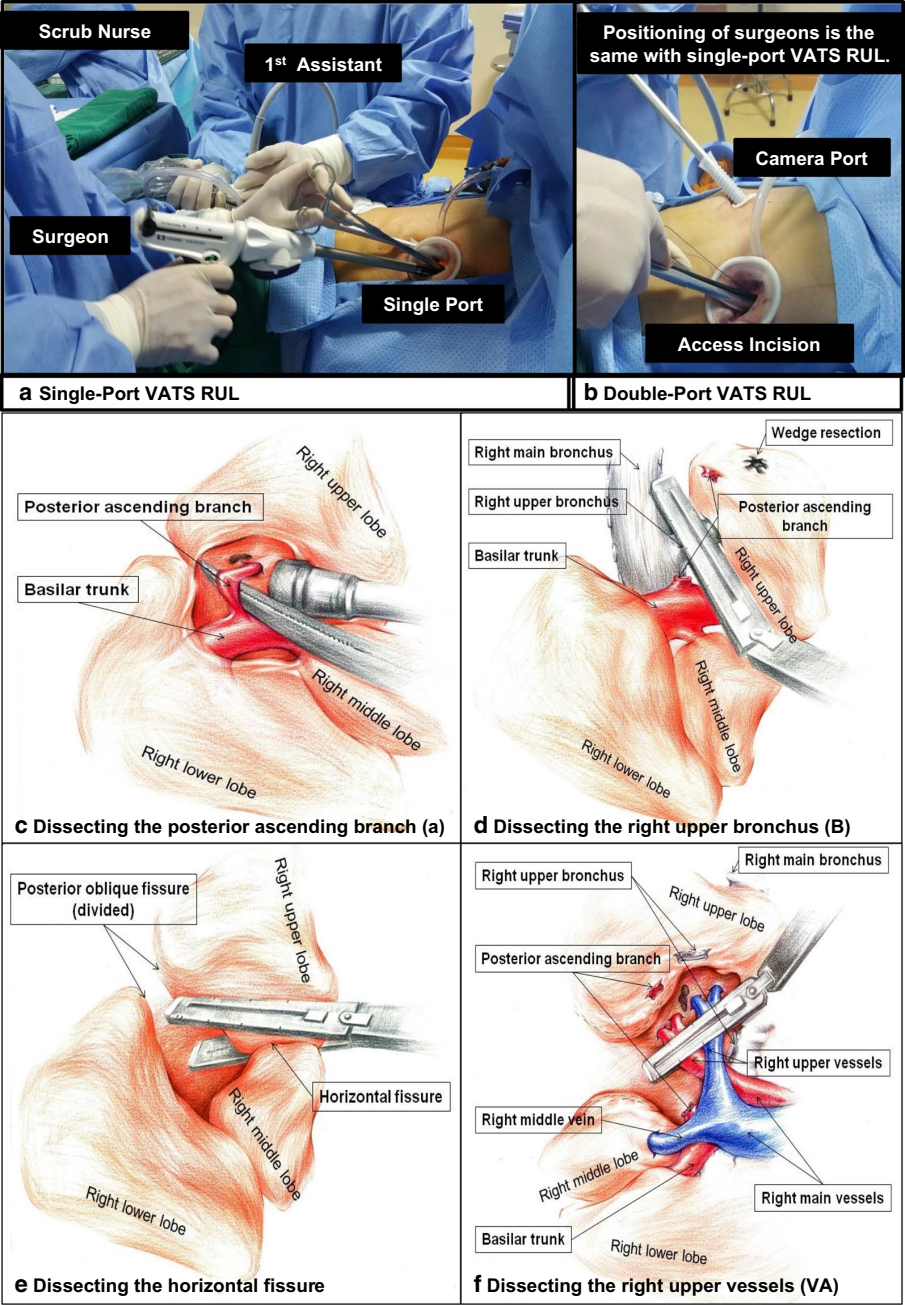
The positioning of surgical and nursing members during the surgery and four key steps of RUL with the aBVA approach. **a** The positioning of surgical and nursing members during the single-port VATS RUL. **b** The positioning of surgical and nursing members during the double-port VATS RUL. **c** Dissecting the posterior ascending arterial branch [a]. **d** Dissecting the right upper bronchus [B]. **e** Dissecting the horizontal fissure. **f** Dissecting the right upper veins and arteries [VA]. *VATS* video-assisted thoracic surgery; *RUL* right upper lobectomy; *aBVA* RUL with the dissecting order of the posterior ascending arterial branch [a], followed by the right upper bronchus [B] and pulmonary vessels [VA]

The chief differences between the two surgical approaches were whether the pulmonary vein was dissected first to mitigate potential micrometastasis and whether the bronchus was dissected before pulmonary vessels. Either two ports or a single port was used during aBVA RUL via VATS (Fig. 1) [26]. Surgeons first dissected the visceral pleura at the crossover of the horizontal fissure and posterior oblique fissure. Interlobar lymph nodes were identified and usually regarded as a landmark to expose the posterior ascending arterial branch (“a” in aBVA). Dissection of the posterior ascending arterial branch allowed better exposure of the right upper bronchus. Second, the anterior, superior, and posterior mediastinal pleura around the hilum were dissociated to facilitate dissecting the right upper bronchus with a stapler. Third, a manual tunnel running through the anterior to the posterior hilum facilitated the division of the fused horizontal fissure. Next, the remaining adhesions around the hilum were freed as much as possible. A stapler was used to dissect the remaining branches of the pulmonary arteries and veins (more details in Additional file 1).

The conventional single-direction procedure of VAB RUL proceeds from the anterior to the posterior site, that is, from the superficial to the deeper site [8]. The right upper vein was first dissociated by a stapler after opening the anterior mediastinal pleura, followed by dissecting the right upper arterial branches. Then, the right upper lobe was distracted toward the inferior and posterior sides for clear exposure of the right upper bronchus. After dissociation of adhesions, the right upper bronchus was dissected. The remaining horizontal and oblique fissures were dissected by staplers through a manual tunnel, as performed with the aBVA approach.

Because the selected patients had indications for RUL, systemic node dissection was applied during both surgical approaches to remove the right upper and lower paratracheal, subcarinal, paraesophageal, and pulmonary ligament lymph nodes. Selective lymph node dissection was only performed on patients undergoing wedge resection, who were excluded in this study.

### Follow-up

After surgery, patients were followed with computed tomography (CT) scans of the chest and upper abdomen every 3 months for up to 2 years and every year thereafter. When chief symptoms causing suspicions of metastases, such as headache, blurred vision, and ostealgia, were reported during the follow-up period, patients underwent brain magnetic resonance imaging (MRI) every 6 months and bone emission computed tomography (ECT) scans every 12 months to detect potential metastases. The last date of follow-up was on 13 November, 2016.

### Statistical analyses

The Statistical Product and Service Solutions (SPSS) software (version 20.0, IBM, Armonk, NY, USA) was used to analyze the data. Categorical variables were analyzed with the Chi square test or Fisher’s exact test. Disease-free survival (DFS) was defined from the date of surgery to the date of disease recurrence or death due to any reason. Overall survival (OS) was calculated from the date of surgery to the date of death. Patients alive at the end of the study were censored at the last follow-up. DFS and OS were estimated using the Kaplan–Meier method and compared between the groups with the log-rank test. A two-sided *P* value of <0.05 was considered statistically significant.

## Results

### General characteristics of patients

**Fig. 2:**
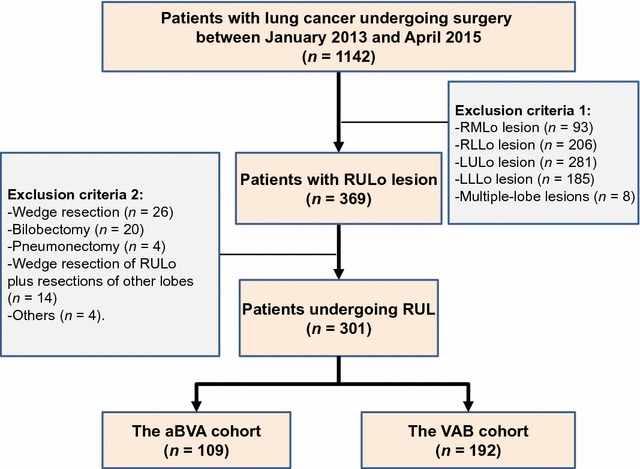
The study flow chart to screen eligible patients undergoing RUL with two different surgical approaches. *RULo* right upper lobe; *RUL* right upper lobectomy; *RMLo* right middle lobe; *RLLo* right lower lobe; *LULo* left upper lobe; *LLLo* left lower lobe; *aBVA* RUL with the dissecting order of the posterior ascending arterial branch [a], followed by the right upper bronchus [B] and pulmonary vessels [VA]; *VAB* RUL with the dissecting order of the right upper pulmonary veins and arteries [VA], followed by the right upper bronchus [B]

A total of 1142 consecutive patients diagnosed with lung cancer were screened retrospectively. A total of 301 patients undergoing VATS RUL were selected: 109 in the aBVA cohort and 192 in the VAB cohort (Fig. 2).

General clinical characteristics were balanced (*P* > 0.05) except for histological subtype (*P* = 0.033) **(**Table 1 **)**. Several confounding factors related to surgical difficulties were identical between the two cohorts (*P* > 0.05) or favored the aBVA procedure with more radical dissection of lymph nodes (*P* < 0.05) (Table 2). There were less dissected lymph node stations but more dissected lymph nodes in the aBVA cohort than in the VAB cohort (both *P* < 0.05). Other factors (maximum tumor diameter, tumor stage, fused fissures) were quite well distributed (*P* > 0.05).

**Table 1:** Comparisons of patients’ clinical characteristics between the aBVA and VAB cohorts

Variable	Total (*n* = 301)	aBVA cohort (*n* = 109)	VAB cohort (*n* = 192)	*P*
Age [years, median (range)]	62 (30–83)	62 (35–81)	62 (30–83)	0.966
Female [cases (%)]	186 (61.8)	65 (59.6)	121 (63.0)	0.561
Smoker [cases (%)]	101 (33.6)	39 (35.8)	62 (32.3)	0.538
Neoadjuvant therapy [cases (%)]	13 (4.3)	4 (3.7)	9 (4.7)	0.775*
Histological subtype [cases (%)]				
Adenocarcinoma	248 (82.4)	98 (89.9)	150 (78.1)	0.033
Squamous cell carcinoma	37 (12.3)	7 (6.4)	30 (15.6)	
Others	16 (5.3)	4 (3.7)	12 (6.3)	
TNM stage [cases (%)]				0.539*
I	209 (69.4)	80 (73.4)	129 (67.2)	
II	42 (14.0)	12 (11.0)	30 (15.6)	
III	44 (14.6)	16 (14.7)	28 (14.6)	
IV	6 (2.0)	1 (0.9)	5 (2.6)	

*aBVA* right upper lobectomy (RUL) with the dissecting order of the posterior ascending arterial branch [a], followed by the right upper bronchus [B] and pulmonary vessels [VA]; *VAB* RUL with the dissecting order of the right upper pulmonary veins and arteries [VA], followed by the right upper bronchus [B]

* Fisher’s exact test

**Table 2:** Comparison of characteristics related to surgical difficulties between the aBVA and VAB cohorts

Variable	Total (*n* = 301)	aBVA cohort (*n* = 109)	VAB cohort (*n* = 192)	*P*
Maximum tumor diameter (cm, mean ± SD)	2.83 ± 1.35	2.72 ± 1.20	2.89 ± 1.42	0.530
T stage [cases (%)]				0.181*
1a	70 (23.3)	24 (22.0)	46 (24.0)	
1b	46 (15.3)	11 (10.1)	35 (18.2)	
2a	139 (46.2)	58 (53.2)	81 (42.2)	
2b	20 (6.6)	5 (4.6)	15 (7.8)	
3	23 (7.6)	9 (8.3)	14 (7.3)	
4	3 (1.0)	2 (1.8)	1 (0.5)	
Incomplete fissure [cases (%)]	94 (31.2)	29 (26.6)	65 (33.9)	0.192
Intrathoracic adhesion [cases (%)]	84 (27.9)	30 (27.5)	54 (28.1)	0.911
Number of dissected LN stations (mean ± SD)	7.13 ± 1.98	7.8 ± 1.92	6.74 ± 1.91	*<0.001*
Number of dissected LNs (mean ± SD)	21.52 ± 9.42	22.95 ± 9.13	20.71 ± 9.51	*0.031*

*aBVA* RUL with the dissecting order of the posterior ascending arterial branch [a], followed by the right upper bronchus [B] and pulmonary vessels [VA]; *VAB* RUL with the dissecting order of the right upper pulmonary veins and arteries [VA], followed by the right upper bronchus [B]; *SD* standard deviation; *LN* lymph node

* Fisher’s exact test

### Operative variables related to surgical feasibility

Comparisons of operative and postoperative outcomes are listed in Table 3. The mean operation time for the aBVA cohort was shorter than that for the VAB cohort (164 vs. 221 min, *P* < 0.001); the mean estimated blood loss was less in the aBVA cohort than in the VAB cohort (92 vs. 141 mL, *P* < 0.001). The aBVA procedure required less staples to dissect the hilar structures than did the conventional VAB approach (5.52 ± 1.85 vs. 6.85 ± 2.29, *P* < 0.001). No conversions to thoracotomy were performed for the aBVA cohort, but were performed on 22 (11.5%) patients in the VAB cohort (*P* < 0.001), indicating the surgical feasibility of aBVA. Eight (36.36%) of 22 intrathoracic conversions to thoracotomy in the VAB cohort were due to VATS-related problems, such as vessel injuries.

**Table 3:** Comparison of operative and postoperative outcomes between the aBVA and VAB cohorts

Variable	Total (*n* = 301)	aBVA cohort (*n* = 109)	VAB cohort (*n* = 192)	*P*
Average operation time (min, mean ± SD)	198.75 ± 74.73	164.08 ± 54.52	221.11 ± 77.55	*<0.001*
Estimated blood loss (mL, mean ± SD)	122.83 ± 145.62	91.74 ± 80.09	141.25 ± 170.68	*<0.001*
Conversion to thoracotomy [cases (%)]	22 (7.31)	0 (0)	22 (11.46)	*<0.001*
Duration of chest drainage (days, mean ± SD)	4.13 ± 4.03	3.55 ± 2.82	4.45 ± 4.55	*0.001*
Postoperative hospitalization (days, mean ± SD)	8.20 ± 6.16	7.73 ± 4.40	8.46 ± 6.96	0.209
Total number of patients developing postoperative complications [cases (%)]	71 (23.6)	24 (22.0)	47 (24.5)	0.629
Procedure-specific complications [cases (%)]^a^	56 (18.6)	18 (16.5)	38 (19.8)	0.482
Prolonged air leakage >7 days	23 (7.6)	7 (6.4)	16 (8.3)	0.549
Serious hemorrhage	17 (5.6)	6 (5.5)	11 (5.7)	0.935
Atelectasis requiring sputum suction via bronchoscopy	14 (4.7)	3 (2.8)	11 (5.7)	0.239
Subcutaneous emphysema	12 (4.0)	4 (1.3)	8 (4.2)	0.752*
Pneumothorax	10 (3.3)	3 (2.8)	7 (3.6)	1.000*
Chylothorax	4 (1.3)	3 (2.8)	1 (0.5)	0.137*
Non-procedure-specific complications [cases (%)]^a^	30 (10.0)	9 (8.3)	21 (10.9)	0.456
Pneumonia	16 (5.3)	7 (6.4)	9 (4.7)	0.519
Atrial fibrillation	6 (2.0)	1 (0.9)	5 (2.6)	0.423*
Pulmonary artery embolism	4 (1.3)	0 (0)	4 (2.1)	0.300*
Stroke	2 (0.7)	1 (0.9)	1 (0.5)	1.000*
Acute myocardial infarction	1 (0.3)	0 (0)	1 (0.5)	1.000*
Other reasons delaying discharge	5 (1.7)	0 (0)	5 (2.6)	0.163*
Surgical costs (10,000 Yuan, mean ± SD)	4.03 ± 0.93	3.85 ± 0.84	4.13 ± 0.96	*0.003*
Hospitalization costs (10,000 Yuan, mean ± SD)	6.89 ± 3.27	6.52 ± 1.71	7.10 ± 3.88	0.176

*aBVA* RUL with the dissecting order of the posterior ascending arterial branch [a], followed by the right upper bronchus [B] and pulmonary vessels [VA]; *VAB* RUL with the dissecting order of the right upper pulmonary veins and arteries [VA], followed by the right upper bronchus [B]; *SD* standard deviation

* Fisher’s exact test. Data of continuous variables are presented as mean ± standard deviation

^a^Multiple complications might occurred in one patient

### Postoperative variables related to recovery

Patients in the aBVA cohort had shorter duration of chest drainage (3.55 vs. 4.45 days, *P* = 0.001) and similar postoperative hospitalization (7.73 vs. 8.46 days, *P* = 0.209) when compared with patients in the VAB cohort. These results favored aBVA for feasible operative procedures and substantial recovery after surgery. The rates of total postoperative complications and procedure-specific complications in the two cohorts were similar (22.0% vs. 24.5%, *P* = 0.629; 16.5% vs. 19.8%, *P* = 0.482) (Table 3). The most frequent procedure-specific complications were air leakage lasting more than 7 days, serious hemorrhage, and atelectasis requiring sputum aspiration by bronchoscopy. The rates of non-procedure-specific complications were also comparable (8.3% vs. 10.9%, *P* = 0.456).

In addition to the aforementioned benefits, patients who underwent the aBVA procedure had less financial burden than those who underwent the VAB procedure (costs of surgery and hospitalization, *P* = 0.003 and *P* = 0.176, respectively) (Table 3).

### Survival benefits

**Fig. 3:**
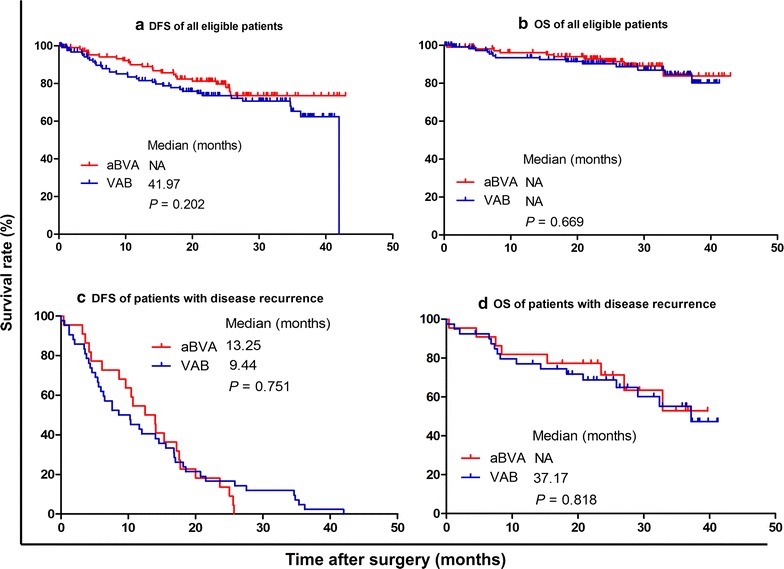
DFS and OS of all eligible patients and patients with disease recurrence between the aBVA and VAB cohorts. **a** DFS of all patients. **b** OS of all patients. **c** DFS of patients with disease recurrence. **d** OS of patients with disease recurrence. *DFS* disease-free survival; *OS* overall survival; *aBVA* RUL with the dissecting order of the posterior ascending arterial branch [a], followed by the right upper bronchus [B] and pulmonary vessels [VA]; *VAB* RUL with the dissecting order of the right upper pulmonary veins and arteries [VA], followed by the right upper bronchus [B]; *NA* not arrived

DFS and OS of the two cohorts were not significantly different (both *P* > 0.05) (Fig. 3a, b). The median DFS for the aBVA cohort was not arrived; that for the VAB cohort was 41.97 months (*P* = 0.202). Among patients with disease recurrence (Fig. 3c, d), the median DFS and OS were still similar in the two cohorts (13.25 vs. 9.44 months, *P* = 0.751; not arrived vs. 37.17 months, *P* = 0.818).

During the follow-up period, 64 (21.3%) patients experienced disease recurrence: 22 in the aBVA cohort and 42 in the VAB cohort (*P* = 0.709) (Table 4). Taking recurrence models into consideration, intrathoracic local recurrence (6.4% vs. 7.8%, *P* > 0.05) and distant metastases intrathoracic local recurrence (10.1% vs. 8.3%, *P* > 0.05) were comparable in both cohorts.

**Table 4:** Comparison of recurrence models in patients with disease recurrence between the aBVA and VAB cohorts

Variable	Total [cases (%)] (*n* = 301)	aBVA cohort [cases (%)] (*n* = 109)	VAB cohort [cases (%)] (*n* = 192)	*P* value
Total	64 (21.3)	22 (20.2)	42 (21.9)	0.709
Intrathoracic local recurrence^a^	22 (7.3)	7 (6.4)	15 (7.8)	0.790
Distant recurrence	27 (9.0)	11 (10.1)	16 (8.3)	0.429
Intrathoracic local recurrence and distant recurrence	5 (1.7)	2 (1.8)	3 (1.6)	1.000*
Unknown	10 (3.3)	2 (0.7)	8 (4.2)	0.472*

*aBVA* RUL with the dissecting order of the posterior ascending arterial branch [a], followed by the right upper bronchus [B] and pulmonary vessels [VA]; *VAB* RUL with the dissecting order of the right upper pulmonary veins and arteries [VA], followed by the right upper bronchus [B]

* Fisher’s exact test

^a^One patient was diagnosed with the progression of multiple primary lesions in each group, which should also be classified as having intrathoracic local recurrence

## Discussion

This study focused on RUL via VATS and demonstrated potential variables that affect surgical feasibility and survival benefits. We identified that the aBVA approach resulted in shorter operation time and less blood loss during surgery, promoted postoperative recovery by reducing the duration of chest drainage, and produced comparable rates of postoperative complications. Although OS and DFS benefits were identical for the selected patients, the aBVA approach may favor intrathoracic local disease control, and the VAB approach may mitigate distant metastases better.

As discussed and depicted in the surgery video for the aBVA procedure (Additional file 1), in situ dissection of the right upper bronchus before dissecting the pulmonary veins and arteries favors intraoperative procedures. It would alleviate residual air storage in the pulmonary alveoli and eliminate non-inflammatory reactions of bronchial mucosa potentially resulting from pulmonary congestion, thereby reducing producible phlegm as well as acquired pneumonia and pulmonary atelectasis. Before the bronchus is dissected, the lobe mostly remains in situ and is not turned over repeatedly, which is different from the VAB procedure. The overall rate of postoperative air leakage would be under control. Furthermore, after dissecting the bronchus, it is more feasible to open the mediastinal pleura proceeding from the posterior towards the superior and anterior sites. This clockwise rotation starts distant from the surgeon and gradually moves closer toward the surgeon, thereby broadening the operative visual field and reducing interferences between surgical instruments.

Traditionally, it was believed that dissecting the pulmonary veins before the pulmonary arteries might reduce disseminations of malignant tumor cells through circulations [27]. Because tumor lesions are repeatedly squeezed during the entire process of surgery, theoretically, there are high risks related to releasing tumor cells into the pulmonary circulation and transferring them to systemic arteries throughout the body, thereby increasing micrometastases. Therefore, interrupting the pulmonary veins before the lobe is repeatedly turned over and compressed would mitigate potential distant metastases. However, previous publications revealed controversial conclusions [19, 22, 28]. Refaely et al. [22] revealed that the sequence of vessel dissection did not interfere and was not a risk factor for tumor recurrence (odds ratio = 1.29, 95% confidence interval 0.73–2.29, *P* = 0.40) based on the data of 279 patients. Another randomized trial conducted by Kozak et al. [28] concluded that the 5-year survival rates of patients with the veins or arteries interrupted first were comparable. However, Sienel et al. [19] determined that 18% (11/62) of patients were confirmed to have detectable tumor cells in the pulmonary veins. This was associated with worse survival benefits, especially for patients with mediastinal lymph node metastases. Therefore, they suggested that the pulmonary veins should be interrupted first to eliminate additional systemic dissemination of tumor cells. In our study, the potential propensity of less distant metastases was consistent with the research of Sienel et al. [19], regardless of the comparable DFS benefits and premature OS results. Further studies comparing the abundance of tumor cells in the pulmonary veins and arteries would provide more solid evidence.

This retrospective study might have some censoring data clouding the interpretation of analyses. Although all RUL procedures via VATS were performed by surgeons with more than 5 years of experience performing VATS, a potential selection bias existed because of the inherent limitations of retrospective studies. However, it has provided thoracic surgeons with insight regarding different orders of dissecting the pulmonary vessels and bronchus, which should be taken into account to determine the surgical feasibility and postoperative recovery. Because it was a retrospective study, the blood samples from the pulmonary veins before surgery were not available, and we could not analyze circulatory tumor cells or compare them with those in the pulmonary arteries. Further studies comparing the abundance of tumor cells in the pulmonary veins and arteries during lobectomies are warranted [29].

## Conclusions

During aBVA RUL via VATS, dissecting the right upper bronchus before turning over the lobe repeatedly and dissecting the pulmonary veins would promote surgical feasibility and achieve comparable postoperative recovery for lung cancer patients. Although survival benefits are comparable, the aBVA approach tends to control local disease recurrences better than the VAB approach. The different orders of dissecting pulmonary structures may have significant importance on improving surgical feasibility. Further research on the effects of different dissecting orders during surgeries on long-term survival benefits of lung cancer patients is warranted.

## Additional file

**Additional file 1.** Surgery video for right upper lobectomy (RUL) with the aBVA approach. This video described RUL with a dissecting order of the posterior ascending arterial branch (a) followed by the right upper bronchus (B) and pulmonary vessels (VA). After intrathoracic exploration, a wedge resection was performed for pathologic diagnosis with frozen sections. When waiting for pathologic reports with highly suspicion of malignancies, surgeons dissociated the posterior ascending arterial branch and hilar lymph nodes. Once primary lung cancer was confirmed, RUL with the aBVA approach was performed via video-assisted thoracic surgery (VATS).
